# Portfolio analysis of NCI-funded policy implementation science grants (fiscal years 2014–2023)

**DOI:** 10.1186/s43058-025-00755-4

**Published:** 2025-07-22

**Authors:** Amira Haidar, Aubrey Villalobos, Cynthia Vinson, Gila Neta

**Affiliations:** Division of Cancer Control and Population Sciences, National Cancer Institute, National Institutes of Health, 9609 Medical Center Dr. , Rockville, MD 20850 USA

**Keywords:** Policy, Implementation science, Cancer control

## Abstract

**Background:**

The dynamic landscape of cancer implementation science (IS) has seen a significant rise in the use of policy as a tool. This analysis explores the multifaceted nature of policy in IS, not only as a directive to be adopted or implemented but also as context that can influence or a strategy that can shape and support effective implementation of cancer control interventions.

**Methods:**

The NIH Query View Report tool was used to identify National Cancer Institute-funded IS grants related to policy between fiscal years 2014–2023. Three coders reviewed the abstracts and specific aims of the grants to ensure a focus on IS, followed by a secondary review to verify the relation to policy. Eligible grants were then coded to determine policy conceptualization methods, level of policy being targeted, alignment with U.S. National Cancer Institute activities, cancer continuum focus, cancer type(s), and cancer content area.

**Results:**

Of the 41 IS grants identified, 14 (34.1%) were included in the analysis. More than half (*n* = 8, 57.1%) of the grants were awarded in FY2020-2023. Most (*n* = 10, 71.4%) grants conceptualized policy as something to implement, 28.6% (*n* = 4) each conceptualized policy as a strategy to use or as something to adopt, and 35.7% (*n* = 5) as context to understand. Ten grants (71.4%) addressed cancer prevention, three grants (21.4%) addressed new innovations, and two grants each (14.3%) addressed expanding access to cancer screenings and supporting cancer survivors and their caregivers. No grants examined policies that address toxic and environmental exposures. Seven grants (50%) addressed the National Cancer Institute activities of increasing access to care.

**Conclusions:**

The NCI funds policy IS across the cancer continuum and in alignment with several National Cancer Institute activities. However, there remain many opportunities to expand policy IS, especially regarding policies that impact cancer diagnosis, treatment, and survivorship and policies related to environmental exposures. Our study highlights key opportunities to conduct research that will inform the adoption, implementation, and use of policies to address the cancer burden in the U.S..

Contributions to the literature

- This study highlights the multifaceted roles of policy within cancer IS—as a directive, context, or strategy—broadening the conceptual framework for how policies can be leveraged to support effective cancer control interventions across the continuum of care.
- This research uncovers underrepresented areas in policy implementation science related to cancer control, like addressing toxic exposures or supporting cancer patients and their caregivers, emphasizing the need for targeted research.
- This study aligns findings with the U.S. National Cancer Institute activities, offering insights to enhance research efforts for cancer outcomes by highlighting critical gaps in addressing cancer prevention, treatment, survivorship, and environmental exposures.

## Background

In pursuit of effective cancer control strategies, the role of policy and implementation science (IS) has become increasingly recognized [1, 2]. As cancer remains a significant public health challenge globally, policymakers and researchers alike are turning their attention to understanding how policy can be leveraged to facilitate the implementation of evidence-based practices across the cancer continuum. As Emmons and colleagues argue, embracing policy implementation science is essential to translating evidence into impactful cancer control policies [3]. Building on this foundation, our study aims to stimulate more systematic consideration of policy implementation within the broader field of implementation science, particularly in the context of cancer control.

The National Cancer Institute (NCI) plays a pivotal role in advancing cancer control research and addressing critical gaps in our understanding of cancer control. Cancer control was established as a top priority in the United States through the enactment of the National Cancer Act in 1971 and underscored by recent White House declaration to end childhood cancer within ten years and the goals outlined in the U.S. National Cancer Plan [4]. The NCI aims to reduce the burden of cancer through collaborative efforts in innovation, policy, and public health initiatives to prevent, screen, diagnose, and treat cancer, and support patients and their caregivers. Recognizing the influence of policy in cancer control interventions and the growing use of IS in the field, understanding the initiatives funded at this intersection holds significant promise for shaping future strategies in combating cancer effectively.

This paper seeks to provide a comprehensive analysis of the research portfolio funded by the NCI at the intersection of policy and IS. This analysis explores the multifaceted nature of policy in IS, not only as a directive to be adopted or implemented but also as context that can influence or a strategy that can shape and support effective implementation of cancer control interventions [2]. By examining the scope of research conducted, we aim to 1) describe key characteristics and trends of policy IS projects in cancer control, 2) identify gaps and opportunities in the research portfolio, and 3) investigate their alignment with NCI efforts.

## Methods

### study Sample Identification

**Fig. 1:**
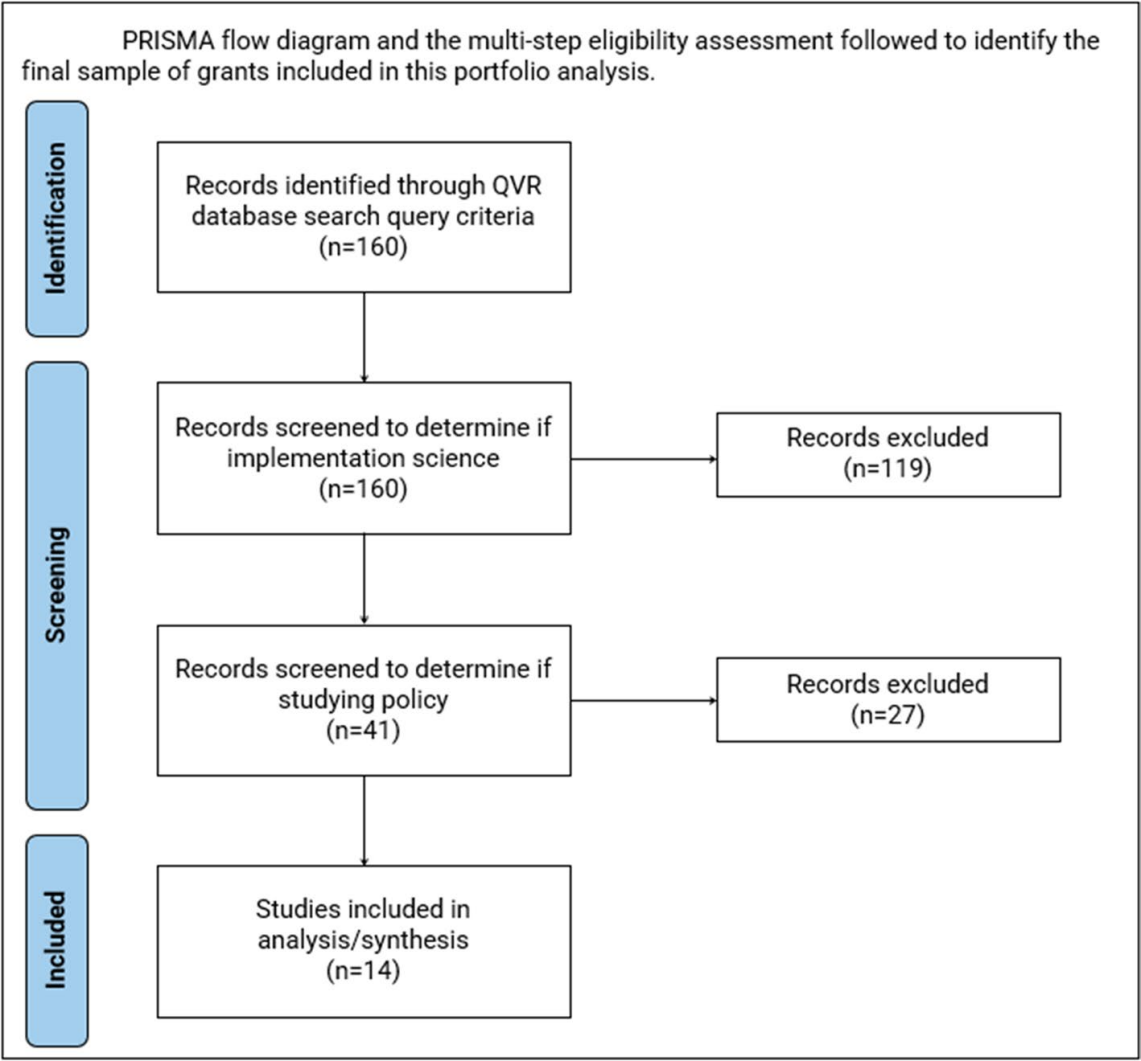
Flow diagram of selection of National Cancer Institute (NCI) policy implementation science grants for analysis

The NIH Query View Report (QVR) tool was used to identify NCI-funded IS grants focused on policy. The search included a keyword search and specific filter criteria including limiting to new and competing continuance grants awarded between the fiscal years 2014–2023 and specific grant mechanisms. Preferred Reporting Items for Systemic Reviews and Meta-Analyses (PRISMA) Statement guidelines were used to document the grant screening process leading to the final sample (Fig. 1) [5]. Finally, multiple team members used iSearch, an NIH internal portfolio analysis platform, to complete the coding process.

### Search strategy

The keyword search comprised two groups of keywords, the first identifying IS grants and the second identifying policy grants. The IS key terms were derived from previously conducted IS portfolio analyses [6, 7]. These key words included: “healthcare delivery”, “cancer care delivery”, “improvement science”, “improvement research”, “quality improvement”, “implementation science”, or “implementation research”. The policy key terms were retrieved from a review on NIH-funded policy dissemination and implementation grants from 2007–2014 [8]. The policy keywords included “policy,” “policies,” “law,” “legal,” “legislation,” “ordinance,” “statute,” “regulation,” “regulatory,” “code,” or “rule.” Using QVR capabilities, a keyword search was applied to the title, abstract, and specific aims to identify the most relevant grants. The search was limited to new and competing continuance grants awarded or completed between the fiscal years 2014 to 2023, with NCI as the administrative center. The grants were then filtered to include only research grant mechanisms U, P, and R series. We excluded training and other capacity building grant mechanisms (i.e., N, F, K T, R25, R13, P30, LRP), and biomedical or bench research.

### Eligibility criteria

Following the initial search, three coders reviewed the abstracts and specific aims pages of the grants to ensure a focus on IS. From the grants identified as IS grants, a secondary review was conducted to verify the focus on policy. This eligibility was informed by Bogenschneider’s definition of policy as: “the development, enactment, and implementation of a plan or course of action carried out through law, rule, code, or other mechanism in the public or private sector” and the Centers for Disease Control and Prevention’s definition of policy as: “a law, regulation, procedure, administrative action, incentive, or voluntary practice of governments and other institutions” [9, 10]. Based on these definitions, the coders reviewed the specific aims pages of the IS grants and included those that explicitly focused on the use of policy or addressed policy as the ‘plan’ or ‘course of action’ in the different forms that it takes. The policy IS grants that were identified were then shared with the IS Team at NCI, who then identified two additional grants from managed funding assignments that were not captured by the search. The remaining grants were used as the analytic sample.

### Codebook development and coding process

The codebook was developed based on a review of other NIH- and NCI-specific grants portfolio analysis codebooks and related publications [6, 7]. The research team also reviewed literature on policy IS to develop codes and definitions that addressed the objectives of the analysis and analyzed the nature of policy IS grants that have been funded. For example, we used the typology from Purtle et al. to categorize the ways in which policy was conceptualized in the studies [2]. The initial codebook developed was then reviewed by the research team and feedback was incorporated. Eligible grants were coded to determine policy conceptualization methods, level and type of policy being targeted, alignment with National Cancer Plan and other NCI activities, cancer continuum focus, cancer type(s), cancer content area, and study characteristics including design and Theory, Model, or Framework used.

Two coders (AH, GN) built the finalized codebook into iSearch (Appendix A) and all four authors coded grants on the platform, with each grant being reviewed by two coders. The coding inconsistencies were subsequently addressed through discussion and an additional review of grant content until consensus was reached.

### Data analysis

Descriptive details of the grants were extracted from QVR and transferred to Microsoft Excel. These details encompassed administrative data such as the year of award and the grant mechanism. The iSearch data were downloaded into Microsoft Excel where frequency and descriptive statistics were calculated to characterize the sample of the grants according to the codebook variables.

## Results

### Grant characteristics

Between fiscal years 2014 and 2023, 14 policy IS grants were funded. Half of these grants were funded through the"Dissemination and Implementation Research in Health"(PAR13-054, PAR16-238, PAR19-275, PAR19-274) funding opportunities (Table 1). Ten grants (71%) were large research projects (e.g., R01, R37, or U01 funding mechanisms). Four grants were exploratory research projects (R21). All projects were U.S.-based.

**Table 1:** Funding characteristics of policy implementation science grants (*N* = 14)

	**Frequency**	**Percent**
**Year Awarded**		
2023	2	14.3
2022	3	21.4
2020	3	21.4
2019	1	7.1
2018	2	14.3
2017	2	14.3
2014	1	7.1
**Funding Mechanism**		
R01	7	50
R21	4	28.6
R37	2	14.3
U01	1	7.1
**Notice of Funding Opportunity**		
Dissemination and Implementation Research in Health (PAR13-054, PAR16-238, PAR19-275, PAR19-274)	7	50
NIH Research Project Grant (Parent R01 Clinical Trial Not Allowed) (PA19-056, PA18-484)	3	21.4
Linking the Provider Recommendation to Adolescent HPV Vaccine Uptake (R01 Clinical Trial Optional) (PAR19-360)	1	7.1
Tobacco Control Policies to Promote Health Equity (R21 Clinical Trial Optional) (PAR20-303)	1	7.1
Traceback Testing: Increasing Identification and Genetic Counseling of Mutation Carriers through Family-based Outreach (U01 Clinical Trial Optional) (PAR18-616)	1	7.1
U.S. Tobacco Control Policies to Reduce Health Disparities (R01) (PAR17-217)	1	7.1

### Cancer study characteristics

The grants were also analyzed for their cancer characteristics (Table 2) such as cancer continuum, type, and content area. Most of the grants (*n* = 11, 78.6%) are focused on cancer prevention. Cancer screening interventions account for 21.4% of the grants, highlighting efforts to detect cancer early. Treatment and survivorship projects are less represented, each with 14.3%. The majority of the grants (*n* = 9, 64.3%) do not specify a particular cancer type, suggesting a broad application of prevention and control strategies across multiple cancer types. Among those that do specify, skin cancer is the most frequently studied (two grants), followed by prostate cancer, ovarian cancer, and HPV-related cancer (one grant each). The grants cover a diverse range of cancer content areas. Three grants (21.4%) focused broadly on cancer prevention and control. HPV vaccination, tobacco control, and UV protection each account for 14.3%. Other areas of focus included nutrition, genomics, rural care delivery, fertility preservation, and de-implementing low-value care (one grant each), highlighting a wide array of research interests and policy priorities within cancer prevention and control.

**Table 2:** Cancer characteristics of the interventions under study in NCI-funded grants (*N* = 14)

	**Frequency**	**Percent**
Cancer Continuum*		
Prevention	11	78.6
Screening	3	21.4
Treatment	2	14.3
Survivorship	2	14.3
**Cancer Type**		
Skin cancer	2	14.3
Prostate cancer	1	7.1
Ovarian cancer	1	7.1
HPV-related cancer	1	7.1
Not specified	9	64.3
**Cancer Content Area**		
General cancer prevention and control	3	21.4
HPV vaccination	2	14.3
Tobacco control	2	14.3
UV protection	2	14.3
Diet	1	7.1
Genomics	1	7.1
Rural care delivery	1	7.1
Fertility preservation	1	7.1
De-implementing low value care	1	7.1

^*^ Not mutually exclusive

### Implementation study characteristics

The analysis of policy concepts, policy levels, policy types, study designs, and theoretical frameworks used in the NCI-funded grants provides several key insights into the focus and methodologies of current research at the intersection of policy and IS (Table 3). Most of the grants (*n* = 10, 71.4%) conceptualize policy as something to implement. Understanding policy as a context was also commonly studied (*n* = 5 grants, 35.7%). Additionally, four grants studied policy as something to adopt and four, policy as a strategy to use. Grants often focused on more than one policy level. Eight grants each focused on organizational and state-level policies. Federal policies are less frequently addressed (*n* = 3, 21.4%), and local policies are the least represented (*n* = 2, 14.3%).

**Table 3:** Implementation study characteristics (*N* = 14)

	Frequency	Percent
Policy Concept*		
Policy as something to implement	10	71.4
Policy as context to understand	5	35.7
Policy as something to adopt	4	28.6
Policy as strategy to use	4	28.6
Policy Level*		
Organizational	8	57.1
State	8	57.1
Federal	3	21.4
Local	2	14.3
**Study Design**		
Non-experimental	10	71.4
Experimental	4	28.6
**Policy Type**		
Public (e.g., Laws, regulations)	7	50
Clinical practice (e.g., guideline)	2	14.3
Health care financing/reimbursement	2	14.3
Health department decision making	2	14.3
Workplace	1	7.1
Theory, Model, or Framework*		
RE-AIM [11]	4	28.6
CFIR [12]	3	21.4
EPIS [13]	2	14.3
Diffusion of Innovations [14]	1	7.1
Framework of public health program capacity for sustainability [15]	1	7.1
Conceptual Framework for Sustainability of Public Health Programs [16]	1	7.1
Theoretical Domains Framework [17]	1	7.1
Dynamic Sustainability Framework [18]	1	7.1
Multiple [Policy] Streams Framework [19]	1	7.1
Analytic Framework for the Explanation of Policy Dismantling [20]	1	7.1
None	2	14.3

^*^ Not mutually exclusive

For policy type, public policies, such as laws and regulations, are the most frequently studied, comprising 50% (*n* = 7) of the grants. Clinical practice guidelines, health care financing/reimbursement, and health department decision-making each account for 14.3%, showing a diverse range of policy types under investigation. Workplace policies are the least studied, with only one grant.

A variety of theories, models, and frameworks are employed across the grants. The Reach Effectiveness Adoption Implementation Maintenance (RE-AIM) framework is the most used (*n* = 4, 28.6%), followed by the Consolidated Framework for Implementation Research (CFIR) (*n* = 3, 21.4%) and Exploration Preparation Implementation Sustainment (EPIS) (*n* = 2, 14.3%). Several others, such as Diffusion of Innovations, various sustainability frameworks, and policy-specific models, each represent 7.1% of the grants. Notably, two projects (14.3%) s do not indicate the use of any specific theory, model, or framework (TMF). For those two projects, we reviewed the full research strategy to confirm that an implementation science TMF was not mentioned. Most of the projects employ non-experimental designs (*n* = 10, 71.4%). Experimental designs are utilized in four (28.6%) of the grants.

### Alignment with national cancer institute activities

The predominant activity addressed by the grants is"Prevent Cancers Before They Start,"which accounts for 71.4% of the total (Table 4). Other activities such as"Develop and Deliver New Innovation"(*n* = 3, 21.4%),"Expand Access to Cancer Screenings"(*n* = 2, 14.3%), and"Support Patients and Caregivers"(*n* = 2, 14.3%) are also represented, though to a lesser extent. Notably, there are no grants focused on"Understand and Prevent Toxic and Environmental Exposures,"highlighting a gap in addressing policy IS on environmental risk factors.

**Table 4:** Alignment with national cancer priorities (*N* = 14)

	**Frequency**	**Percent**
**NCI Activities**		
Prevent Cancer	10	71.4
Develop and Deliver New Innovation	3	21.4
Expand Access to Cancer Screenings	2	14.3
Support Patients and Caregivers	2	14.3
Understand and Prevent Toxic and Environmental Exposures	0	0
National Cancer Plan*		
Prevent Cancer	10	71.4
Eliminate Inequities	7	50
Detect Cancers Early	3	21.4
Deliver Optimal Care	3	21.4
Develop Effective Treatments	0	0
Engage Every Person	0	0
Maximize Data Utility	0	0
Optimize the Workforce	0	0

^*^ Not mutually exclusive

Alignment with the National Cancer Plan reveals similar trends."Prevent Cancer"is the most frequently addressed objective, with 71.4% of grants targeting this area."Detect Cancers Early"and"Deliver Optimal Care"are each targeted by 21.4% (*n* = 3 each) of the grants. No grants address"Develop Effective Treatments,""Engage Every Person,"and"Maximize Data Utility."

## Discussion

The analysis of NCI-funded grants reveals that policy-oriented IS remains a relatively limited portfolio but has more than doubled in recent years. This doubling appears to be a consistent trend when we compare our findings to a previously published policy-focused portfolio analysis that reviewed grants between 2007 and 2014 [8]. Purtle et al. identified six policy-focused IS grants during the 8-year period between 2007–2014, whereas we found 14 grants over the subsequent 10-year period (2014–2023), roughly doubling the rate of grants. The existing grants span across the cancer care continuum and encompass a wide variety of topics in cancer control, indicating a broad, albeit small, scope of funded projects that are aligned with national cancer control priorities [4].

Since 2019, NCI has supported a policy IS action group through the Consortium for Cancer Implementation Science, the goal of which is to develop strategies to support the IS community in advancing research in cancer control policy implementation. Action group members have produced several webinars, resources, and academic articles over the years in service of this goal [21].

In an effort to stimulate further policy IS, NCI recently funded four 1-year administrative supplements (NOT-CA-23–044) [22]. Awardees are advancing this science in diverse projects including a participatory dynamic simulation modeling study of hospital community benefits (e.g., medical financial assistance) policies, examining the impact of state immunization information systems policies on HPV vaccination rates, evaluating a dissemination strategy to expand clinical practice guideline-concordant genetic risk assessment to underserved racial and geographic populations, and examining the dissemination related to a state law that aims to improve access to cancer care and clinical trials for Medicaid beneficiaries. This is one small step to fill the gap and move the field forward, but greater investment is needed.

The grants reviewed in this portfolio analysis cover various stages of the cancer care continuum, from prevention to survivorship, aligning with National Cancer Institute activities. A substantial proportion of grants are aimed at preventing cancers before they start (71.4%), with fewer focused on cancer screening, treatment, and survivorship, demonstrating both a holistic approach to cancer control and an opportunity for additional research. Among the cancer prevention projects, a range of policy levers are explored including using technology to scale up occupational sun protection programs, the use of local land use and zoning policies’ influence on tobacco retail density, federal child nutrition assistance policies to improve diet, the use of academic and public health department partnerships, and legal solutions to facilitate genetic testing and cascade screening. Among projects focused on supporting cancer patients, investigators focused on policies to support de-implementation of low value care, the impact of rural hospital payment and delivery reform on cancer surgery, and strategies to improve implementation of the health benefits mandate for fertility preservation.

A significant gap identified in the current funding landscape is the lack of focus on understanding policies to prevent or mitigate exposures to toxic and environmental carcinogens. Despite the critical role that environmental factors and toxic exposures play in cancer risk, no grants specifically targeted this priority area. This gap represents a crucial opportunity for future policy-oriented research to address the impact of environmental carcinogens, a critical component of cancer prevention and control. Neta, Martin, and Collman (2022) outline opportunities for environmental health researchers to leverage implementation science in their work to develop, disseminate, implement, and sustain interventions, including guidelines and policies for mitigating exposures and reducing cancer risk [23].

Big changes in cancer care necessitate robust policy interventions. Evidence-based policies are essential for driving these transformations and ensuring that scientific advancements translate into practical, widespread improvements in cancer prevention, treatment, and survivorship. The limited number of policy-focused grants suggests that there is substantial room for growth in this area. By framing these gaps as opportunities, the field can consider policy research that leverages scientific evidence to inform and shape effective cancer control policies. Chriqui et al. (2023) recommend ways the IS field can evolve to more quickly and fully achieve the promise of policy research to improve health outcomes [24]. If successful, expansion and application of policy IS could accelerate work to address structural and social drivers of health for the advancement of health for all patients [25].

The identified gaps in environmental health and policy research highlight significant opportunities for future investigation. Expanding research in these areas can address critical aspects of cancer prevention and control that are currently underrepresented. Environmental health research, in particular, can provide valuable insights into the prevention of cancer through the identification and mitigation of environmental risk factors. Additionally, a stronger focus on policy research can ensure that the latest scientific discoveries are implemented through effective, evidence-based policies that improve cancer outcomes across populations.

This analysis is not without limitations. Although we used a range of search terms to identify policy implementation science grants, it is possible that our search was not exhaustive. However, these terms have been used in other similar published portfolio analyses and likely captured the vast majority of relevant grants. Further, we queried program staff to identify additional grants that were not originally captured. Also, because we restricted our analysis to NCI-funded grants, the 14 grants included in our analysis may not reflect the broader policy implementation science field outside of cancer-related research.

## Conclusions

The NCI has supported IS projects addressing policy across the cancer continuum and in alignment with several U.S. National Cancer Institute activities. However, there remain many opportunities to expand the field, especially regarding policies that impact cancer diagnosis, treatment, and survivorship and policies related to environmental exposures. This study highlights key opportunities to conduct research that will inform the adoption, implementation, and use of policies to address the cancer burden in the U.S.

## Data Availability

Full copies of the grants from which these data were generated are considered confidential per federal regulations and NIH grant policies and are not available upon request or available in the public domain.

## Appendix A

### Policy Portfolio Analysis Codebook

1. In which way is policy conceptualized in the implementation study? Select all that apply. (Variable: Policy_Concept)
  - Policy as something to adopt
  - Policy as something to implement
  - Policy as context to understand
  - Policy as strategy to use
2. Does the study address one of the five National Cancer Institute activity areas? (Variable: NCI_Activities)
  - Expand Access to Cancer Screenings
  - Understand and Prevent Toxic and Environmental Exposures
  - Prevent Cancers
  - Develop and Deliver New Innovation
  - Support Patients and Caregivers
3. Does the study address any of the 8 National Cancer Plan goals? (Variable: National Cancer Plan)
  - Prevent Cancer
  - Detect Cancers Early
  - Develop Effective Treatments
  - Eliminate Inequities
  - Deliver Optimal Care
  - Engage Every Person
  - Maximize Data Utility
  - Optimize the Workforce
4. What level is the policy targeting? (Variable: Policy Level)
  - Organizational
  - Local (e.g. municipal, county)
  - State
  - Federal
  - International
5. What type of policy is addressed in the study? (Variable: Policy Type)
  - Clinical practice (e.g., guidelines)
  - Health care financing/reimbursement
  - Public (e.g., laws, regulations)
  - Workplace
  - Other
6. Where is the study focused along the cancer control continuum? Select all that apply. (Variable: Cancer Continuum)
  - Prevention
  - Screening
  - Diagnosis
  - Treatment
  - Survivorship
7. What cancer type is the focus of the study? Select one. (Variable: Cancer Site)
  - Bladder Cancer
  - Breast Cancer
  - Cervical Cancer
  - Colorectal Cancer
  - Endometrial
  - Kidney Cancer
  - Leukemia
  - Liver Cancer
  - Lymphoma
  - Myeloma
  - Ovarian Cancer
  - Prostate Cancer
  - Skin Cancer
  - Testicular Cancer
  - Thyroid Cancer
  - Other
  - Not specified
8. What cancer content area does the study focus on? Select all that apply unless encompassed by comprehensive content area (e.g., survivorship care). (Variable: Content Area).
  - Alcohol use
  - Cancer screening
  - Chemoprevention
  - Decision making
  - Diet
  - Genomics
  - HBV/HCV
  - HPV testing
  - HPV Vaccination
  - Mental Health
  - Obesity
  - Palliative care
  - Physical activity
  - Surveillance
  - Tobacco control
  - UV protection
  - Other/Not specified
9. What type of study design is used? Select all that apply. (Variable: Policy study design).
  - Experimental
  - Non-experimental
  - Quasi-experimental
10. Theory, model, or framework used in the study? Select all that apply. (Variable: TMF).
  - ADAPT-ITT
  - Conceptual Framework for Sustainability of Public Health Programs (Dearing)
  - Conceptual Model of Evidence-based Practice Implementation in Public Service Sectors (Aarons)
  - Consolidated Framework for Implementation Research
  - Diffusion of Innovations
  - Diffusion of Innovations in Service Organizations (Greenhalgh)
  - Dynamic Sustainability Framework
  - EPIS
  - Framework by Shediac-Rizkallah and Bone
  - Framework of public health program capacity for sustainability (Schell, Luke)
  - Interactive Systems Framework
  - Organizational Theory of Innovation Implementation
  - PARIHS
  - PRECEDE/PROCEED
  - PRISM
  - Proctor’s Implementation Outcomes Framework
  - Program Sustainability Assessment Tool
  - Other
  - Not specified/none

## Abbreviations

IS: Implementation science
CFIR: Consolidated framework for implementation research
EPIS: Exploration preparation implementation sustainment
HPV: Human papillomavirus
NCI: National Cancer Institute
PRISMA: Preferred Reporting Items for Systemic Reviews and Meta-Analyses
RE-AIM: Reach Effectiveness Adoption Implementation Maintenance
UV: Ultraviolet
